# Chiral Imprinting on Inorganic Nanoparticles for Enantioselective Surface Recognition

**DOI:** 10.1002/smll.202507490

**Published:** 2025-10-14

**Authors:** Susanna Tinello, Mélanie Emery, Markus Niederberger

**Affiliations:** ^1^ Department of Materials Laboratory for Multifunctional Materials ETH Zurich Vladimir‐Prelog‐Weg 5 Zurich 8093 Switzerland

**Keywords:** surface functionalization, chirality, enantioselectivity, nanoparticles, sol–gel processes

## Abstract

Chiral semiconductor nanoparticles, especially metal oxides, hold great potential for selective photocatalysis and enantiomer separation. Chirality in such systems can emerge during nanoparticle nucleation and growth, leading to optical activity in the absorption range of the material. A key aspect of this phenomenon is the transfer of chirality from a chiral organic molecule interacting with the nanoparticle surface to the inorganic core. In this study, threoninol is used as a chiral ligand to induce chirality in titanium dioxide (TiO_2_) nanoparticles, and its binding mode to the metal oxide is investigated through nuclear magnetic resonance (NMR) spectroscopy. The analysis reveals that threoninol interacts with the TiO_2_ surface primarily via hydrogen bonding through its hydroxyl groups. To determine whether a chiral imprint is retained, the ligand is fully removed using ultraviolet (UV) irradiation, and the nanoparticles are subsequently re‐exposed to a racemic mixture of threoninol. Remarkably, the nanoparticles selectively re‐bind the enantiomer used in the original synthesis. This provides evidence for the presence of enantioselective active sites on the TiO_2_ surface, suggesting that the chiral ligand imprints chirality through structural features. Although the precise mechanism of chiral imprinting remains speculative, the findings demonstrate that inorganic nanomaterials can be endowed with enantioselective properties.

## Graphical abstract

Chiral imprinting on titanium dioxide (TiO_2_) nanoparticles is achieved by introducing a chiral ligand during nucleation and growth. After ligand removal by ultraviolet (UV) irradiation, enantioselectivity is demonstrated via refunctionalization in a racemic mixture, where the surface preferentially binds the original enantiomer. These results show that inorganic nanomaterials can exhibit enantioselective properties.

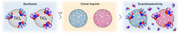

## 1 Introduction

Chirality, the property of a structure that cannot be superimposed on its mirror image, is a fundamental concept of biological systems and plays a crucial role in molecular interactions. In pharmaceuticals, enantiomers of chiral drugs often exhibit different biological activities, affecting their efficacy and safety. While research on chirality has traditionally focused on organic molecules, there has been increasing attention on extending chirality to inorganic nanostructures. These materials, which exhibit size‐dependent optical and catalytic properties, offer exciting new opportunities for chiral recognition and enantioselective interactions,^[^ ^1^ ^]^ asymmetric catalysis,^[^ ^2^ ^]^ and biosensing.^[^ ^3^ ^]^

Several mechanisms can induce chirality in inorganic nanomaterials.^[^ ^4^, ^5^ ^]^ It can result from a) the intrinsic chiral crystal structure,^[^ ^6^, ^7^, ^8^ ^]^ b) chiral nanoparticle shape,^[^ ^9^, ^10^ ^]^ c) assembly of nanoparticles in chiral superstructures,^[^ ^11^ ^]^ d) lattice distortions and displacements of atoms on the inorganic surface in response to interaction with surface ligands,^[^ ^12^, ^13^ ^]^ e) asymmetric arrangement of stabilizing ligands on the surface of an inorganic core,^[^ ^14^ ^]^ f) an electric field from surface ligands that generates an asymmetric polarization pattern in the nanoparticles, as seen in plasmonic systems,^[^ ^15^, ^16^ ^]^ or g) polarized light‐induced chirality.^[^ ^17^, ^18^ ^]^ Among these mechanisms, the chirality induced by the adsorption of a chiral ligand on an inorganic surface is particularly intriguing, as it suggests that the ligand can distort the surface atomic arrangement, potentially leaving behind a chiral imprint even after ligand removal.^[^ ^12^, ^19^ ^]^ This phenomenon has significant implications for enantioselective catalysis and chiral separations, as it could allow inorganic surfaces to selectively interact with one enantiomer over the other.

Unlike chiral nanostructures with inherently twisted shapes, ligand‐induced chirality relies on molecular interactions to create a chiral mark on an achiral inorganic material. If a nanoparticle retains enantioselective properties even after ligand removal, it suggests that the surface itself has undergone a structural reorganization. This concept, often referred to as chiral imprinting, raises important questions such as: *Can inorganic nanoparticles retain chiral recognition properties after removal of the chiral ligand? Can this property be exploited for enantioselective catalysis or chiral separations?*

While significant progress has been made in understanding chiral metal nanocrystals,^[^ ^20^, ^21^ ^]^ research on chiral semiconductor nanoparticles remains relatively unexplored. Early work by Moloney et al.^[^ ^12^ ^]^ on CdS quantum dots showed that the binding of chiral penicillamine ligands could induce chiral defects on the nanoparticle surface, a hypothesis later supported by density functional theory (DFT) calculations.^[^ ^19^ ^]^ Further research demonstrated the formation of chiral defects on the surface of other quantum dots, such as CdTe, using various chiral ligands.^[^ ^22^, ^23^ ^]^ Nakashima et al.^[^ ^24^ ^]^ were the first to observe that CdTe nanoparticles retained their optical activity after a ligand exchange with an achiral ligand. They reported this result as a “chiral memory effect”. This phenomenon was also reported by Mukhina et al.,^[^ ^1^ ^]^ where the optical activity of CdS nanotetrapods was retained after the chiral ligand was removed from the surface by reverse phase transfer. Fourier transform infrared (FT‐IR) spectroscopy confirmed that no chiral ligand remained on the surface. In contrast, elemental analysis in Nakashima's work suggested that the chiral ligand was only partially exchanged (92%).^[^ ^24^ ^]^ These contradictions raise important questions about the mechanism behind chiral imprinting and whether a completely ligand‐free nanoparticle can retain enantioselective properties.

Based on these considerations, titanium dioxide (TiO_2_) emerges as an attractive material for the development of chiral semiconductor nanoparticles due to its well‐known photocatalytic properties,^[^ ^25^ ^]^ stability, and broad applications in sensing,^[^ ^26^ ^]^ photochemical water splitting,^[^ ^27^ ^]^ and photovoltaics.^[^ ^28^ ^]^ If chiral TiO_2_ nanoparticles with enantioselective surface sites could be synthesized, it would open new possibilities for applications in chiral separation, as an inorganic alternative to traditional chiral stationary phases, and in asymmetric catalysis, especially in photocatalytic reactions where chirality at the active sites could induce enantioselectivity.

The successful synthesis of chiral TiO_2_ nanoparticles with enantioselective surface sites could unlock a new frontier in materials science—paving the way for groundbreaking applications in chiral separation as a robust inorganic alternative to conventional chiral stationary phases, and in asymmetric catalysis, particularly in photocatalytic processes where surface chirality could drive enantioselective reactivity.

In fact, Cleary et al.^[^ ^29^ ^]^ found that TiO_2_ nanoparticles capped with diphenylethylenediamine during the synthesis showed no optical activity after heat treatment, leading them to conclude that no chiral imprint remained after complete removal of the ligands. Liu et al.^[^ ^30^ ^]^ reported a different approach, namely the synthesis of chiral TiO_2_ nanofibers, in which the chirality resulted from the arrangement of TiO_2_ nanoparticles into a double‐helix structure. However, this system relied on macroscopic assembly rather than intrinsic chiral imprinting at the atomic level.^[^ ^31^ ^]^ These studies highlight a critical gap in the field: while ligand‐induced chiral effects have been observed in some semiconductor nanomaterials, no conclusive evidence for a persistent chiral imprint on ligand‐free TiO_2_ nanoparticles has been reported. If such an imprint exists, it could have significant implications for the development of enantioselective catalysts and chiral recognition materials.

In this study, we provide evidence for a chiral imprint on the surface of TiO_2_ nanoparticles. Specifically, we investigated whether chirality could be imprinted during the nucleation and growth of TiO_2_ prepared by non‐aqueous sol–gel synthesis^[^ ^32^, ^33^, ^34^ ^]^ in the presence of l/d‐threoninol. Importantly, threoninol binds to the TiO_2_ surface without inhibiting nanoparticle formation, inducing chiroptical activity while simultaneously stabilizing the nanoparticles in aqueous dispersions. To demonstrate the presence of a chiral imprint, the chiral ligand was removed by ultraviolet light treatment (UV‐treatment), and the complete removal was evaluated by nuclear magnetic resonance (NMR) spectroscopy. The UV‐treated nanoparticles were then redispersed in a racemic mixture of l/d‐threoninol and characterized by circular dichroism (CD) spectroscopy. Although the nanoparticles show no detectable optical activity after removal of the ligand, they retain the remarkable ability to selectively bind the enantiomer introduced during their synthesis. This strongly suggests the presence of enantioselective active sites on the surface of TiO_2_ nanoparticles. While the exact mechanisms underlying this phenomenon remain unclear and difficult to determine experimentally, ligand‐induced atomic rearrangements are a likely explanation. Regardless, these intriguing results open up exciting avenues for future research into the origins of surface‐based enantioselectivity.

## 2 Results and Discussion

TiO_2_ nanoparticles were synthesized by a non‐aqueous sol–gel method in benzyl alcohol, using metal halides as molecular precursors. To investigate the potential chiral imprinting on the nanoparticles, l/d‐threoninol enantiomers were introduced as chiral ligands during the synthesis by varying the molar ratio of Ti^4+^ precursor to threoninol, with four different ratios tested: 12:1, 10:1, 5:1, and 2.5:1. Each synthesis was carried out separately for both l and d enantiomers of threoninol.

X‐ray diffraction (XRD) analysis confirmed the formation of the anatase polymorph of titania (Figure S1, Supporting Information). The (101) reflection exhibited progressive broadening with increasing ligand concentration, indicating that threoninol plays a role in nanoparticle growth (Figure S2, Supporting Information). Higher ligand concentration likely leads to a more crowded surface, limiting particle growth and resulting in smaller crystallites. Crystallite sizes, calculated from the (101) reflection using Scherrer's equation, were consistent with the transmission electron microscopy (TEM) results (Figure S3, Supporting Information), showing sizes ranging from 3.4 to 6.5 nm, with larger particles corresponding to lower ligand concentrations (Table S1, Supporting Information).

The TEM image (Figure S3, Supporting Information) revealed a tendency for the primary TiO_2_ nanocrystals to undergo oriented attachment, a phenomenon previously observed by Niederberger et al.^[^ ^35^ ^]^ in TiO_2_ synthesized with trizma. Since both threoninol and trizma are amino alcohol molecules, it is plausible that threoninol similarly facilitates oriented attachment by acting as a molecular assembler.

### 2.1 Effect of l/d‐Threoninol on the Chiroptical Activity of TiO_2_

**Figure 1:**
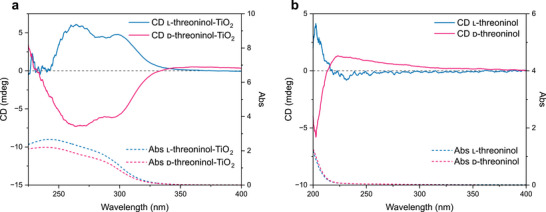
a) CD spectra (solid lines) and UV–vis spectra (dashed lines) of l‐threoninol‐functionalized TiO_2_ NPs (blue) and d‐threoninol‐functionalized TiO_2_ NPs (magenta) with a Ti‐to‐threoninol molar ratio of 5:1. b) CD spectra (solid lines) and UV–vis spectra (dashed lines) of l‐threoninol (blue) and d‐threoninol (magenta) solutions.

Titania nanoparticles functionalized with l‐ or d‐threoninol exhibited chiroptical activity, as evidenced by mirror‐image circular dichroism (CD) signals in the spectral region between 230 and 340 nm corresponding to TiO_2_ absorption (**Figure** **1**a). In contrast, free l/d‐threoninol showed chiroptical activity only in the 200–215 nm range (Figure 1b). This shift suggests that the observed chiroptical activity in threoninol‐functionalized TiO_2_ may result from a ligand‐induced effect, where the highest occupied molecular orbitals (HOMOs) of the chiral ligand overlap with the valence band (VB) states of TiO_2_.^[^ ^13^, ^36^, ^37^ ^]^ The shape and intensity of the CD spectra for the functionalized nanoparticles can be influenced by several factors, including the binding mode of the ligand to TiO_2_, and its spatial arrangement and concentration on the nanoparticle surface.^[^ ^5^, ^14^ ^]^ Notably, the CD spectra of l‐ and d‐threoninol (Figure 1b) are not perfectly symmetrical, which may be attributed to differences in enantiomeric purity (97% for l‐threoninol and 95% for d‐threoninol, as detailed in the Experimental Section). The presence of minor impurities in the starting materials could contribute to deviations from ideal enantiomeric symmetry during surface functionalization. A similar asymmetry is also observed in the CD spectra of the functionalized TiO_2_ (Figure 1a), where the d‐threoninol–functionalized TiO_2_ exhibits slightly positive signals beyond 335 nm, an effect that becomes more pronounced in the corresponding g‐factor spectra (Figure S4, Supporting Information). As shown in Figure S4 (Supporting Information), the g‐factor values lie in the range of 10^−4^, which is characteristic of spherical semiconductor nanomaterials.^[^ ^38^ ^]^ Moreover, for both l‐ and d‐threoninol–functionalized samples, we observe an increase in g‐factor values with increasing threoninol concentration, suggesting a correlation between ligand presence and chiroptical activity (Figure S5, Supporting Information).^[^ ^39^, ^40^ ^]^ Interestingly, the g‐factor values for the d‐threoninol‐functionalized TiO_2_ are consistently higher than those of the l‐threoninol counterparts (Figure S5, Supporting Information). This discrepancy could arise from differences in the purity of the starting materials, or it may suggest that d‐threoninol has a stronger binding affinity to the TiO_2_ surface, resulting in greater surface coverage and thus enhanced chiroptical response.^[^ ^41^, ^42^ ^]^

As a control, TiO_2_ was also synthesized in the absence of threoninol, and no chiroptical activity was observed in this sample (Figure S6, Supporting Information).

### 2.2 Investigation of Threoninol Binding Modes by NMR Spectroscopy

To investigate the binding mode of threoninol to the surface of titania, ^1^H nuclear magnetic resonance (^1^H NMR) spectroscopy was performed on nanoparticles functionalized with different molar ratios of l‐ or d‐threoninol.

**Figure 2:**
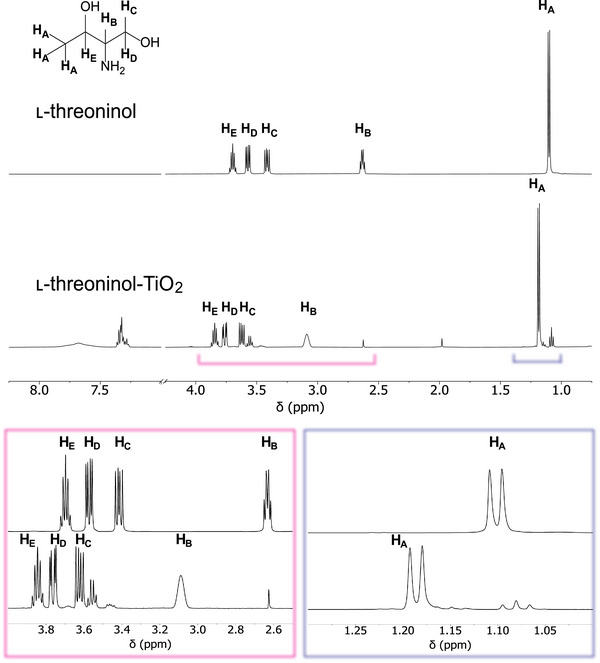
^1^H NMR spectra of l‐threoninol (top), and l‐threoninol‐functionalized TiO_2_ (bottom) at Ti‐to‐threoninol molar ratio of 5:1. Zoomed‐in regions: 2.5–4.0 ppm (pink, left) and 1.0–1.3 ppm (violet, right).

The ^1^H NMR spectrum of free threoninol showed well‐defined signals (**Figure** **2**): a doublet at 1.10 ppm (3 H, methyl group, C1), a quintet at 3.70 ppm (H, C2), a quartet at 2.63 ppm (H, C3), and two doublets of doublets at 3.42 and 3.57 ppm (2 H, C4). In contrast, threoninol‐functionalized TiO_2_ nanoparticles showed downfield‐shifted and broadened threoninol peaks, indicating ligand interaction with the titania surface.^[^ ^43^ ^]^ The downfield shift remained constant over all tested precursor‐to‐ligand ratios, indicating that threoninol interacts consistently with the titania surface regardless of concentration (Figure S7a, Supporting Information). The peak broadening could be attributed to ligand adsorption on the surface of TiO_2_ NPs, which slows molecular tumbling and reduces spin–spin relaxation (T_2_).^[^ ^43^, ^44^ ^]^ Moreover, the absence of distinct peaks corresponding to free ligands suggests that ligand adsorption occurs in a fast chemical exchange regime, where bound and unbound ligand states rapidly interconvert. Thus, the observed signal represents an averaged state between bound and free molecules.^[^ ^43^, ^45^ ^]^

In addition to threoninol, ^1^H NMR spectra of functionalized nanoparticles revealed the presence of benzyl alcohol (7.32 ppm) and ethanol impurities (1.08 and 3.56 ppm), which are likely to be synthesis residues. A broad peak at 7.68 ppm, which was absent in both the pure ligand and unmodified TiO_2_, was consistently observed in the spectra of functionalized nanoparticles (Figure S7b, Supporting Information). To determine its origin, ^1^H NMR measurements were repeated under acidic conditions (pH 2), where the exchange rate between the hydroxyl groups and D_2_O was altered, verifying that the peak corresponds to the hydroxyl groups of threoninol (Figure S8, Supporting Information). The broad peak at 7.68 ppm also appeared in acidified l‐threoninol‐functionalized TiO_2_ but was not present in TiO_2_ synthesized without threoninol (Figure S8b, Supporting Information). This suggests that the hydroxyl groups of the ligand dynamically interact with the TiO_2_ surface, probably through hydrogen bonding. Further evidence for this interaction comes from the integration analysis, where the peak intensity was 1.5 instead of 2, supporting a dynamic exchange between free and surface‐bound hydroxyl groups. This hypothesis was further supported by diffusion‐ordered spectroscopy (DOSY) experiments. The experimental diffusion coefficient for free l‐threoninol acidified to pH 2 was measured as 6.96 × 10^−10^ m^2^ sec^−1^ (Figure S9, Supporting Information). Small organic molecules such as threoninol typically exhibit diffusion coefficients ≈10^−9^ m^2^ sec^−1^,^[^ ^46^ ^]^ suggesting that under acidic conditions, threoninol forms a molecular network in solution, possibly through hydrogen bonding or ionic interactions likely involving its protonated amino group and chloride ions. Interestingly, for titania functionalized with l‐threoninol at a Ti‐to‐threoninol molar ratio of 2.5:1, a slightly faster diffusion coefficient (7.90 × 10^−10^ m^2^ sec^−1^) was observed (Figure S9, Supporting Information). This suggests that the presence of titania nanoparticles may disrupt intermolecular interactions between threoninol molecules in solution. Indirectly, this observation indicates that the interaction between threoninol and TiO_2_ is relatively weak, leading to the observed fast exchange regime.

These findings suggest that threoninol primarily interacts with TiO_2_ nanoparticles through its hydroxyl groups, forming hydrogen bonds with surface sites. This interaction could play a crucial role in the chiral imprinting of the material, where the presence of the ligand induces enantioselective surface properties in the nanoparticles.

### 2.3 Chiral Imprint on TiO_2_ Nanoparticle After Ligand Removal

The key question in developing chiral materials is whether inorganic nanoparticles will remain chiral even if the chiral organic ligands have been removed from the surface. Such an imprinting effect has not been proven beyond doubt, nor has any potential application been demonstrated. In fact, Cleary et al.^[^ ^29^ ^]^ have previously suggested that CD activity in chiral TiO_2_ is due solely to ligand‐induced effects and not to a chiral surface imprint. However, their study used a thermal treatment at 300 °C for 1 h, which could have altered the crystalline structure and surface atoms of titania, potentially erasing any surface chirality. To prevent phase transitions and structural changes, we used an alternative ultraviolet light (UV) treatment for 24 h to remove the chiral ligand while preserving the integrity of the TiO_2_ surface.

To evaluate the efficiency of the UV treatment, the nanoparticles were analyzed by Fourier‐transform infrared – attenuated total reflectance (FTIR‐ATR) spectroscopy (Figure S10, Supporting Information). Comparison of spectra of UV‐treated TiO_2_, TiO_2_ synthesized without chiral ligands, and free ligand confirmed that organic residues were effectively removed at all molar ratios. To further verify these results, we employed ^1^H NMR spectroscopy, which offers higher sensitivity than FTIR and enables the detection of trace amounts of residual organic species. ^1^H NMR analysis (Figure S11, Supporting Information) supported the FTIR finding, except for the Ti‐to‐threoninol molar ratio of 2.5:1, where residual ligand peaks remained, suggesting that prolonged UV exposure may be required for complete removal. However, at the Ti‐to‐threoninol molar ratios of 5:1, 10:1, and 12:1, the absence of characteristic hydrogen peaks associated with the ligand confirmed successful ligand removal.

To investigate whether a chiral imprint persisted on the TiO_2_ surface, a refunctionalization experiment was performed: UV‐treated nanoparticles were dispersed in a racemic mixture of threoninol to determine if the surface retained enantioselectivity. To the best of our knowledge, this type of experiment has never been carried out before and could form the basis for numerous applications. Although there have been studies on ligand substitution by achiral molecules and the nanoparticles retained optical activity,^[^ ^1^, ^24^ ^]^ there is always uncertainty in these approaches as to whether the observed optical activity is due to chiral defects on the surface or residual chiral ligands that were not fully replaced. Our approach aimed first at minimizing these uncertainties by ensuring complete ligand removal, and second by exposing the nanoparticles to a racemic mixture to determine whether one enantiomer selectively interacted with the nanoparticles, providing indirect evidence of chiral imprinting.

To estimate the potential surface coverage, it was necessary to make assumptions about the binding mode of threoninol to titania nanoparticles. The binding mode of polyhydroxy compounds on metal oxides remains underexplored, and in the case of titania, mainly restricted to the rutile phase.^[^ ^47^ ^]^ Based on our pre‐UV‐treatment NMR results, we propose that threoninol interacts by the so‐called bridge bonding mode, that is, each OH group is coordinated to one titanium atom.^[^ ^35^, ^48^, ^49^ ^]^ However, at the experimental pH (≈9.6), interactions involving the amino group cannot be excluded. Due to geometric constraints, it is reasonable to assume that the ligand interacts with the surface through two of its functional groups. To estimate the number of available surface sites, we applied the equation reported by Rajh et al.,^[^ ^50^ ^]^ which allowed us to approximate the molar concentration of surface titanium atoms ([Ti_surf_]) as: [Ti_surf_] = [TiO_2_]·12.5/*d*, with [TiO_2_] being the molar concentration of titania and *d* being the diameter of the particles in angstroms. Based on the assumption that one enantiomer covers the entire nanoparticle surface while the other remains in solution, a 1:1 titanium‐to‐ligand molar ratio was chosen.

**Figure 3:**
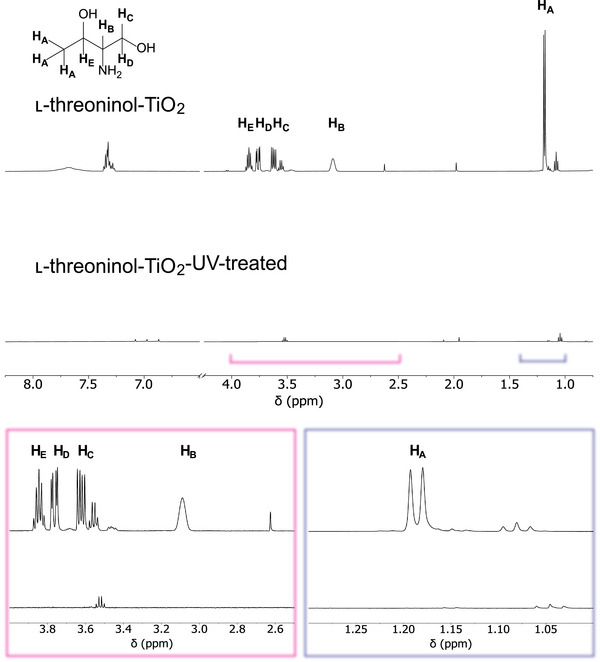
^1^H NMR spectra of l‐threoninol‐functionalized TiO_2_ at a Ti‐to‐threoninol molar ratio of 5:1 before UV‐treatment (top), and after UV‐treatment (bottom). Zoomed‐in regions: 2.5–4.0 ppm (pink, left) and 1.0–1.3 ppm (violet, right).

Refunctionalization experiments were performed on the UV‐treated TiO_2_ synthesized at a Ti‐to‐threoninol molar ratio of 5:1, as ^1^H NMR confirmed complete ligand removal in this case (**Figure** **3**). Furthermore, this ratio was chosen because it provides a higher number of potential surface sites for chiral imprinting compared to the TiO_2_ samples synthesized at a Ti‐to‐threoninol molar ratio of 10:1 and 12:1.

**Figure 4:**
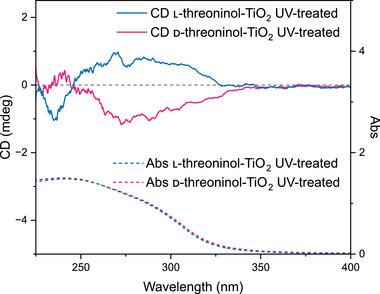
CD spectra (solid lines) and UV–vis spectra (dashed lines) of UV‐treated TiO_2_ nanoparticles initially synthesized with l‐threoninol (blue) and d‐threoninol (magenta) with a Ti‐to threoninol molar ratio of 5:1 after refunctionalization.

After refunctionalization, CD spectra (**Figure** **4**) revealed a broad signal between 245 and 330 nm, corresponding to the same wavelength region where CD activity was observed before ligand removal. Most importantly, nanoparticles originally synthesized with l‐threoninol showed an L signal, while those synthesized with d‐threoninol displayed a D signal. This confirms the presence of a chiral imprint on the TiO_2_ surface after ligand removal, which resulted in the selective binding of the enantiomer that was used during the synthesis.

Notably, the shape of the CD signal changed after refunctionalization. The double peak observed before the ligand removal was no longer present, and a single peak was detected instead. The reduction in chiroptical intensity and the altered spectral shape could be attributed to several factors, including changes in the ligand binding geometry after refunctionalization, nanoparticle aggregation after UV treatment, or incomplete enantioselectivity in ligand rebinding. The presence of rapid chemical exchange between bound and unbound states, as indicated by DOSY NMR experiments, may also contribute to the altered CD response. In addition, dynamic light scattering (DLS) data suggested that the nanoparticles aggregated after UV treatment, reducing the surface area available for ligand interaction and potentially weakening the chiroptical signal (Table S2, Supporting Information).

^1^H NMR analysis was performed after refunctionalization (Figure S12, Supporting Information), demonstrating that threoninol is clearly present, and the characteristic downfield shifting and broadening of the peaks remain, indicating ongoing interaction between threoninol and the TiO_2_ surface. Notably, the broad peak at 7.68 ppm, previously associated with hydrogen bonding between threoninol and TiO_2_, is no longer observed. This disappearance may be due to rapid dynamic exchange processes on the surface, which render this peak undetectable under the experimental conditions. In addition, the pH conditions after synthesis and after refunctionalization were different: the initial synthesis is highly acidic due to the use of TiCl_4_, whereas the refunctionalization occurs under more basic conditions (pH ≈9.6). Given this pH shift, as mentioned before, additional interactions involving the amino group of threoninol cannot be excluded.

Since the pH of the refunctionalization experiment was ≈9.6, a reference sample of UV‐treated TiO_2_ was stabilized at the same pH with sodium hydroxide. No CD signal was detected over the entire wavelength range (Figure S13, Supporting Information). It is important to note that the UV‐treated nanoparticles were not stable in neutral aqueous solution. Although the addition of NaOH may alter the TiO_2_ surface, ^1^H NMR analysis confirmed the complete removal of threoninol. Therefore, we believe that the CD measurement shown in Figure S13 (Supporting Information) reliably reflects the optical properties of TiO_2_ nanoparticles after UV treatment.

An interesting aspect to consider is the role of the hydrogen bond, which can range in strength from 5 to 150 kJ mol^−1^.^[^ ^51^ ^]^ This study showed that the strength of the hydrogen bonds was sufficient to facilitate the interaction between threoninol and the titania surface, inducing chiral imprinting, but not strong enough to prevent dynamic exchange, as shown in the DOSY experiments. This suggests that weak and non‐covalent chemical interactions are sufficient to induce chiral imprinting on an inorganic surface. In addition, the timing of surface functionalization plays a crucial role. Chiral imprinting only occurs if the chiral ligand is already present during synthesis, i.e., during particle formation.

## 3 Conclusion

In this study, we present the synthesis of crystalline and chiral TiO_2_ nanoparticles using a non‐aqueous sol–gel approach with l‐ or d‐threoninol serving as chiral ligands. CD spectroscopy confirmed the chiroptical activity of the functionalized nanoparticles, showing distinct mirror‐image signals for the l‐ and d‐threoninol‐functionalized TiO_2_ attributed to ligand‐induced effects at the nanoparticle surface.

^1^H NMR identified hydrogen bonding via hydroxyl groups as the primary interaction between the ligand and the nanoparticle surface. Complementary DOSY NMR indicated a fast exchange regime that favors the free ligand state. The measured g‐factors were consistent with those typically observed for semiconductor nanoparticles and increased with ligand concentrations, reinforcing the hypothesis that the chiroptical response is driven by surface interactions. A key finding of this work is the demonstration of chiral surface imprinting. Following UV‐induced removal of the chiral ligand, the TiO_2_ nanoparticles were found to selectively re‐bind the enantiomer initially used in their synthesis, providing clear evidence for the formation of enantioselective surface sites. Although CD measurements showed no residual optical activity after ligand removal, the successful refunctionalization in a racemic mixture highlights retained chiral selectivity, indicating that the origin of chirality lies in the surface defects rather than in inherent optical properties. This finding challenges the conventional perspective of chirality as an exclusively molecular property, though the precise mechanism behind this nanoscale phenomenon remains unresolved. Computational studies that examine the ligand–surface interactions, the resulting surface distortions, and the structural evolution after ligand removal are expected to offer deeper insights into the mechanism of chiral imprinting. Furthermore, potential differences in binding affinities between the two enantiomers on distinct TiO_2_ crystal facets could be investigated.

While these open questions present exciting opportunities for further investigation, our results provide the first evidence that purely inorganic nanomaterials can exhibit enantioselective behavior. This discovery paves the way for future applications in asymmetric catalysis, chiral sensing, and enantiomer recognition enabled by successful chiral imprinting on inorganic surfaces.

## Conflict of Interest

The authors declare no conflict of interest.

## Supporting information



Supporting Information

## Data Availability

The data that support the findings of this study are available from the corresponding author upon reasonable request.
