# Supplementary material for: Chiral Imprinting on Inorganic Nanoparticles for Enantioselective Surface Recognition
Source: Small. 2025 Oct 14;21(47):e07490. doi: 10.1002/smll.202507490 (PMC12658927; doi:10.1002/smll.202507490)
Supplement: Supplementary file 1 — Supporting Information [file SMLL-21-e07490-s001.docx]

**Supplementary Information**

**Chiral Imprinting on Inorganic Nanoparticles for Enantioselective Surface Recognition**

Susanna Tinello[a], Mélanie Emery[a], Markus Niederberger[a]

[a] Laboratory for Multifunctional Materials, Department of Materials, ETH Zurich, Vladimir-Prelog-Weg 5, 8093 Zurich, Switzerland

**Contents**

Experimental Section 2

Characterization 5

Results and Discussion 6

References 17

# **Experimental Section**

**Chemicals and Materials**

Titanium tetrachloride (99.9% trace metal basis), anhydrous benzyl alcohol (puriss., > 99.0%), ethanol (absolute 99.8% for analysis), and ethyl acetate (puriss., > 99.7%) were purchased from Sigma-Aldrich. Heptane (fraction) was purchased from Thommen-Furler AG. l-threoninol (puriss., 97%), and d-threoninol (puriss., 95%) were acquired from Fluorochem. All chemicals were used without further purification.

**Synthesis of l/d-threoninol-functionalized TiO_2_ Nanoparticles**

The synthesis route developed by Niederberger et al.^[1, 2]^ was used and adapted to synthesize titania nanoparticles capped with l/d-threoninol, at four different titanium-to-threoninol molar ratios: 12:1, 10:1, 5:1 and 2.5:1. These molar ratios were investigated separately for the l- and d-forms of threoninol.

In the first step of the synthesis, the ligand was dissolved in benzyl alcohol (40 mL) in a 100 mL round-bottom-flask, with the mass of the ligand adjusted according to the molar ratio being tested. In the second step of the synthesis, titanium tetrachloride (2 mL, 18 mmol) was added dropwise to ice-cooled ethanol (6.25 mL, 107 mmol) in another 50 mL round-bottom flask under constant stirring at 200 rpm. The addition rate was adjusted so that the released hydrochloric acid fume continuously redissolved in cold ethanol instead of escaping the flask. After the addition, the greenish-yellow viscous solution was stirred for another 5 min. The resulting yellow ethanolic precursor solution was slowly added to the benzyl alcohol-threoninol mixture under vigorous stirring at room temperature. The mixture was then heated to 120 °C in a preheated oil bath for 2 hours under continuous stirring at 500 rpm. During this period, the reaction solution gradually changed from clear yellow to translucent and eventually to milky, indicating the formation of titania nanoparticles. The reaction mixture was finally cooled to room temperature.

To precipitate the particles, 24 mL aliquots of the reaction solution were mixed with ethyl acetate (7 mL) and heptane (14 mL). The white precipitate was collected by centrifugation for 10 min at 4000 rpm and washed two times with ethyl acetate (35 mL) and subsequently three times with heptane (35 mL). For each washing step, the wet precipitate was mixed with fresh solvent, shaken vigorously, and centrifuged for 3 min at 4000 rpm, before the clear supernatant was discarded.

After the last washing step, TiO_2_ nanoparticles were either dispersed in Milli-Q water or dried to get a powder. To obtain colloidal solutions, the particles from one aliquot were suspended in heptane (30 mL), followed by the addition of Milli-Q water (7 mL) to extract the nanoparticles. The system was stirred gently to maintain the resulting two-phase separation. The functionalized nanoparticles transferred from the heptane phase to the aqueous phase. The aqueous phase containing the TiO_2_ nanoparticles was separated with a syringe and stored in a vial. The nanoparticle powders were obtained by vacuum drying at 50 °C for 24 hours. They were then ground with an agate mortar and pestle into a fine powder and stored in a vial.

**UV-treatment of l/d-threoninol-functionalized TiO_2_ Nanoparticles**

l/d-threoninol-functionalized TiO_2_ nanoparticle powders were irradiated with ultraviolet (UV) light in air using a Hönle UVACUBE2000 to remove the organic matter from their surface. The UV box was equipped with a mercury UV lamp with an arc power output of 2000 W, and the internal temperature did not exceed 60 °C. The standard experimental procedure involved loading threoninol-functionalized TiO_2_ powder into a petri dish. The powder was flattened inside the petri dish to ensure homogeneous irradiation. The petri dish was placed inside the Hönle UVACUBE2000 on a stainless steel laboratory lifting frame covered with aluminium foil to reflect light. The platform is lifted, so that the petri dish was closer to the UV lamp. The nanoparticles were UV-irradiated in air for 3-hour intervals, with a total irradiation time of 24 hours. After each 3-hour interval, the powder was thoroughly mixed to ensure even irradiation of the entire surface.

**Enantioselective Refunctionalization of UV-treated TiO_2_ Nanoparticles**

The l- and d-threoninol powders were dried under vacuum for three days to remove moisture, ensuring accurate mass measurements for the preparation of the racemic mixture. The dried powders were then transferred to a glovebox and stored in separate vials. Based on the assumption that one enantiomer covers the entire nanoparticle surface while the other remains in solution, a 1:1 titanium-to-ligand molar ratio was chosen. We propose that threoninol interacts by the so-called bridge bonding mode, where each OH group is coordinated to one titanium atom. To estimate the number of available surface sites, we applied the equation reported by Rajh et al.^[3]^, which allowed us to approximate the molar concentration of surface titanium atoms ([Ti_surf_]) as: [Ti_surf_] = [TiO_2_]·12.5/d, with [TiO_2_] being the molar concentration of titania and d being the diameter of the particles in angstroms. Comprehensive calculations are outlined in the following section (Section 1).

Stock solutions of each enantiomer were prepared, resulting in final concentrations of 192 mM for l-threoninol (puriss., 97%) and 196 mM for d-threoninol (puriss., 95%). From these stock solutions, 100 μL of each was diluted with 21.8 mL of Milli-Q water to reach a final volume of 22 mL. A 2 mL aliquot was taken from each racemic mixture to assess its quality by circular dichroism (CD) spectroscopy.

To test the enantioselective binding of threoninol, 10 mg of UV-treated TiO_2_ nanoparticles (functionalized with l- or d-threoninol at a Ti-to-threoninol molar ratio of 5:1) were ground, weighed, and added to 20 mL of racemic mixtures at a final concentration of 1764 µM. The suspensions were stirred at room temperature for 24 hours, allowing the nanoparticles to interact with the racemic threoninol mixture. After this step, 5 mL of each mixture was transferred into a centrifuge tube and centrifuged at 4000 rpm for 20 minutes. A 2 mL aliquot of the supernatant was collected and stored in a vial for further analysis.

**Section 1**

Approximation of the number of moles of surface sites on the titanium dioxide nanoparticles:

$$m_{{TiO}_{2}}=10 mg$$

$$V_{tot}=20 mL$$

$$n_{{TiO}_{2}}=\frac{m}{MW}=\frac{10\cdot{10}^{-3}}{79.866}=1.25\cdot{10}^{-4} mol$$

$$C_{{TiO}_{2}}=\frac{n}{V}=\frac{1.25\cdot{10}^{-4}}{20\cdot{10}^{-3}}=6.26\cdot{10}^{-3} M$$

$$\left[ Ti \right]_{surf}=\frac{[{TiO}_{2}]\cdot12.5}{d}=\frac{6.26\cdot{10}^{-3}\cdot12.5}{4.2\cdot10}=1.86\cdot{10}^{-3} M$$

$$n_{{Ti}_{surf}}=C\cdot V=\left[ Ti \right]_{surf}\cdot20\cdot{10}^{-3}=3.73\cdot{10}^{-5} mol$$

The diameter d was calculated from the average of the two crystal sizes of the TiO_2_ nanoparticles obtained with the l- and d-forms after UV treatment.

Calculation of the required mass of the ligand, taking into account that the ligand binds to two different titanium centers via its two alcohol groups:

$$n_{enantiomer}=\frac{n_{{Ti}_{surf}}}{2}=\frac{3.73\cdot{10}^{-5}}{2}=1.86\cdot{10}^{-5} mol$$

$$m_{enantiomer}=MW\cdot n=105.14\cdot1.86\cdot{10}^{-5}=1.96\cdot{10}^{-3}=1.96 mg$$

Taking into account the different purities of the two enantiomers:

$$m_{adjusted L-threoninol}=\frac{1.96}{0.97}=2.02 mg$$

$$n_{L-threoninol}=\frac{2.02\cdot{10}^{-3}}{105.14}=1.92\cdot{10}^{-5} mol$$

$$C_{L-threoninol}=\frac{1.92\cdot{10}^{-5}}{20\cdot{10}^{-3}}=9.60\cdot{10}^{-4} M$$

$$m_{adjusted D-threoninol}=\frac{1.96}{0.95}=2.06 mg$$

$$n_{D-threoninol}=\frac{2.06\cdot{10}^{-3}}{105.14}=1.96\cdot{10}^{-5} mol$$

$$C_{D-threoninol}=\frac{1.96\cdot{10}^{-5}}{20\cdot{10}^{-3}}=9.81\cdot{10}^{-4} M$$

Preparation of the l- and d-threoninol stock solutions:

$$C_{1}\cdot V_{1}=C_{2}\cdot V_{2}$$

$$C_{L-threoninol}=\frac{9.60\cdot{10}^{-4}\cdot20}{100\cdot{10}^{-3}}=192 mM$$

$$C_{D-threoninol}=\frac{9.81\cdot{10}^{-4}\cdot20}{100\cdot{10}^{-3}}=196 mM$$

# **Characterization**

*Powder X-ray diffraction (XRD)*. XRD measurements were performed on a PANalytical X’Pert PRO diffractometer with Cu K_α1_ radiation (45 kV, 40 mA). The diffractogram were collected over a 2θ range of 15-80° with a step size of 0.05°.

*Circular Dichroism Spectroscopy (CD)*. CD measurements were performed using a J-815 CD Spectropolarimeter (Jasco). The spectra were measured at 20 °C in quartz cuvettes with a path length of 5 mm. Kuhn’s dissymmetry factor (g-factor) allows the conversion of circular dichroism and UV absorbance spectra into a dimensionless g-factor spectrum and is defined by the following equation:^[4]^

$$g=\frac{A_{L}-A_{R}}{A}$$

*^1^H Nuclear Magnetic Resonance Spectroscopy (^1^H NMR)*. ^1^H NMR spectra were acquired on a Bruker Avance III HD spectrometer at 11.7 T (500 MHz) equipped with a BBO Prodigy cryoprobe and processed using MestReNova. Spectra were recorded using the WATERGATE W5 pulse sequence with gradients and a double echo to obtain 1D ^1^H NMR spectra.^[5, 6]^ Samples were prepared at a concentration of 100 mM in 90% Milli-Q water and 10% D_2_O.

*Diffusion Ordered Spectroscopy (DOSY)*. DOSY experiments were performed on a Bruker Avance III HD spectrometer at 11.7 T (500 MHz) equipped with a PABBO probe with a 50 G/cm maximum Z-gradient. The DOSY method reported by Jerschow and Mueller,^[7]^ which uses a double stimulated echo sequence with two spoil gradients, was employed. The diffusion time (Δ) was set to 250 ms, and the gradient pulse duration (δ) to 1.5 ms. Diffusion coefficients were determined by fitting the signal decay of the threoninol methyl group to the Stejskal-Tanner equation:^[8]^

$$y\left( x \right)=I_{0}\cdot e^{(-D\cdot\left( 2\pi\cdot\gamma\cdot G\cdot\delta\right)^{2}\cdot\Delta)}$$

where 𝐼_𝑜_ represents the initial signal intensity, D is the diffusion coefficient, 𝛾 is the gyromagnetic ratio, and G represents the gradient strength. Measurements were performed on pure l-threoninol and l-threoninol-functionalized TiO_2_ at a Ti-to-threoninol molar ratio of 2.5:1, both prepared at a concentration of 100 mM and acidified to pH 2 with hydrochloric acid. Moreover, the TiO_2_ sample was analyzed at the same concentration without acidification.

*Fourier-Transform Infrared Spectroscopy (FTIR)*. FTIR spectra were collected using a Bruker Alpha spectrometer equipped with a diamond crystal for Attenuated Total Reflectance (ATR) measurements.

*Dynamic Light Scattering (DLS)*. DLS measurements were performed using a Zetasizer Nano ZS (Malvern) instrument.

*High-Resolution Transmission Electron Microscopy (HRTEM)*. HRTEM analyses were performed on an FEI Talos F200X instrument operated at 200 kV.

# **Results and Discussion**


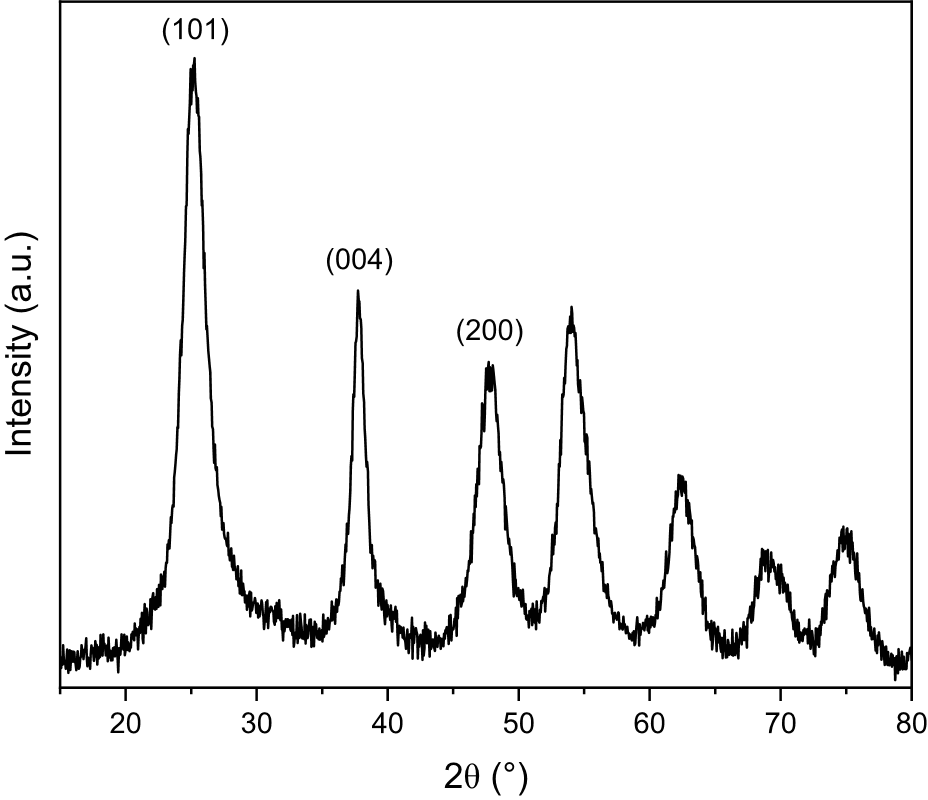


Figure S1. Powder XRD pattern of the anatase TiO_2_ nanoparticles functionalized with l-threoninol with a Ti-to-threoninol molar ratio of 5:1.


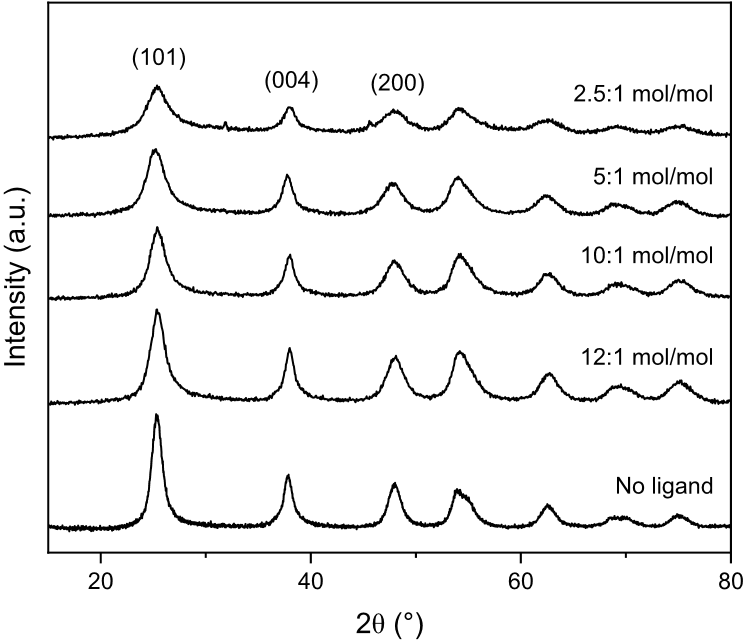


Figure S2. Powder XRD patterns of anatase TiO_2_ nanoparticles functionalized with l-threoninol at different Ti-to-threoninol molar ratios, as well as TiO_2_ synthesized without threoninol for comparison.


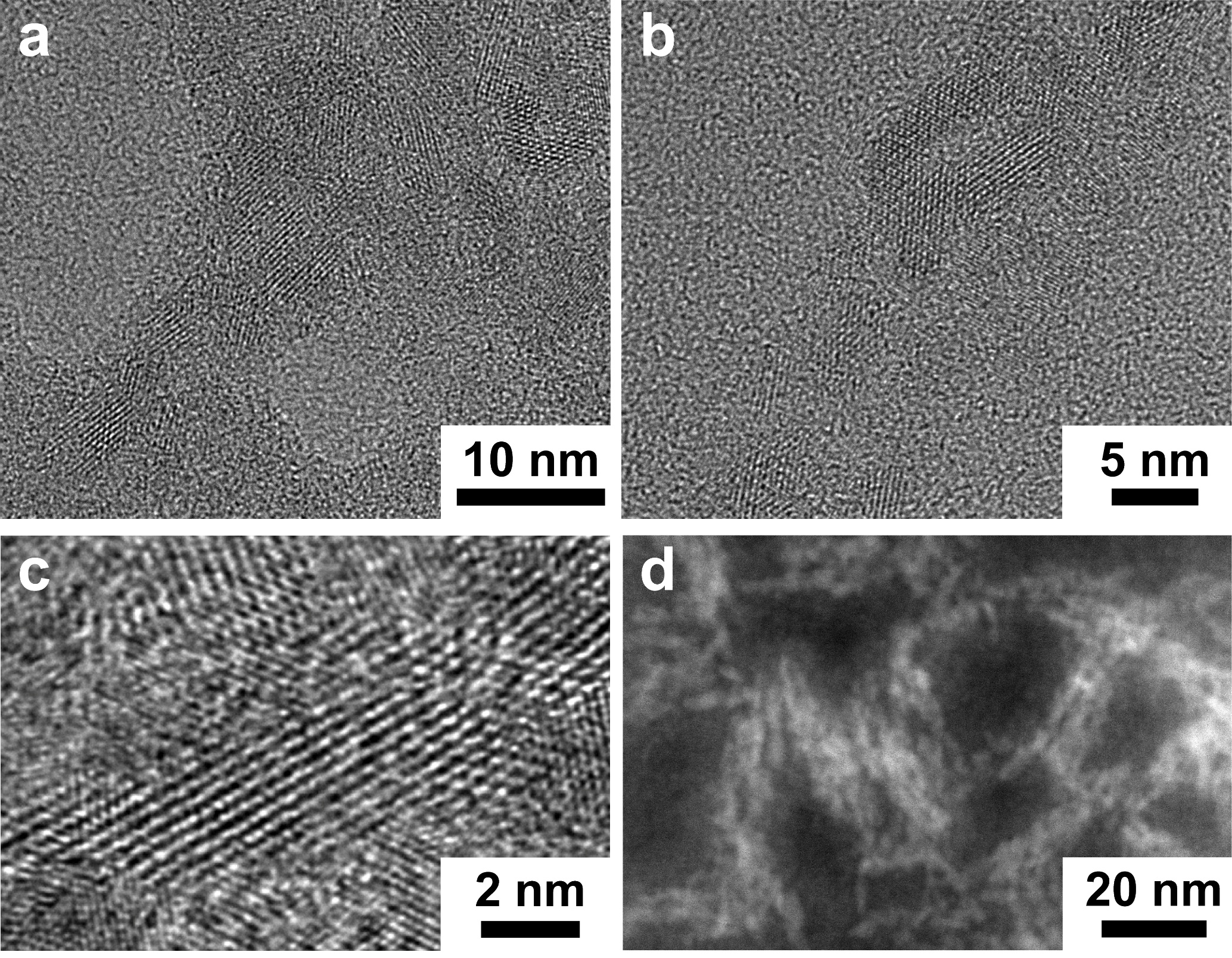


Figure S3. (a-c) TEM and (d) STEM images of l-threoninol functionalized TiO_2_ nanoparticles.

Table S1. Crystallite sizes calculated for TiO_2_ NPs functionalized with l- and d-threoninol at different Ti-to-threoninol molar ratios, as well as TiO_2_ synthesized without threoninol for comparison. The crystallite sizes were determined using the Scherrer equation based on the (101) reflection of the anatase phase.


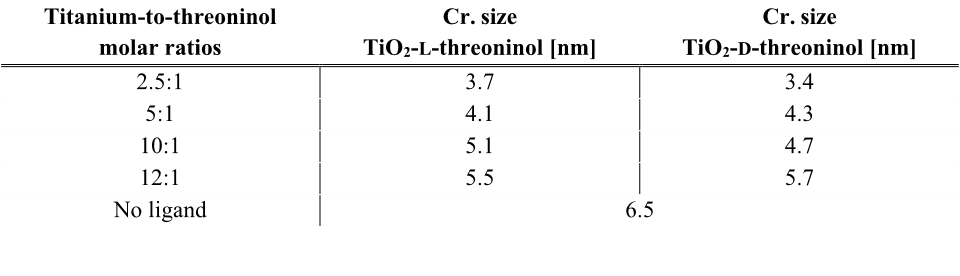


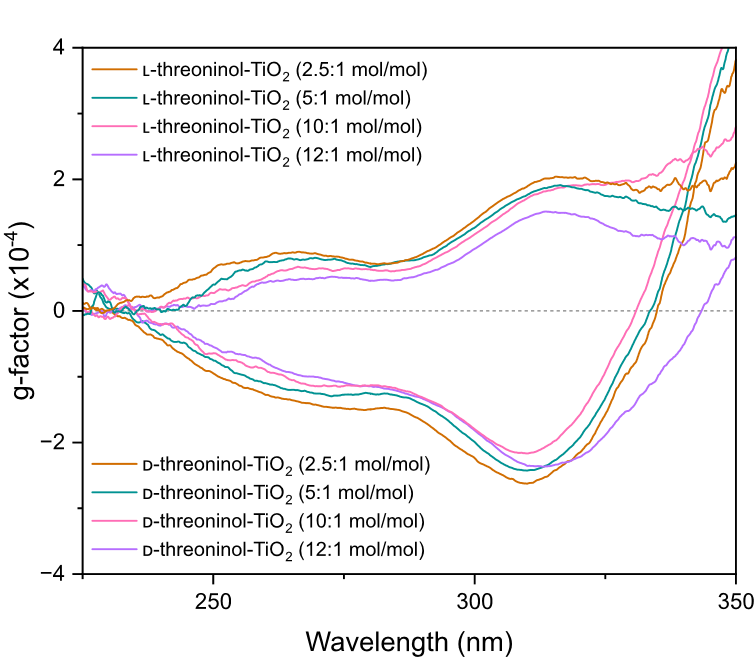


Figure S4. G-factor graphs of threoninol-functionalized TiO_2_ with varying Ti-to-threoninol molar ratios (2.5:1, 5:1, 10:1, 12:1).


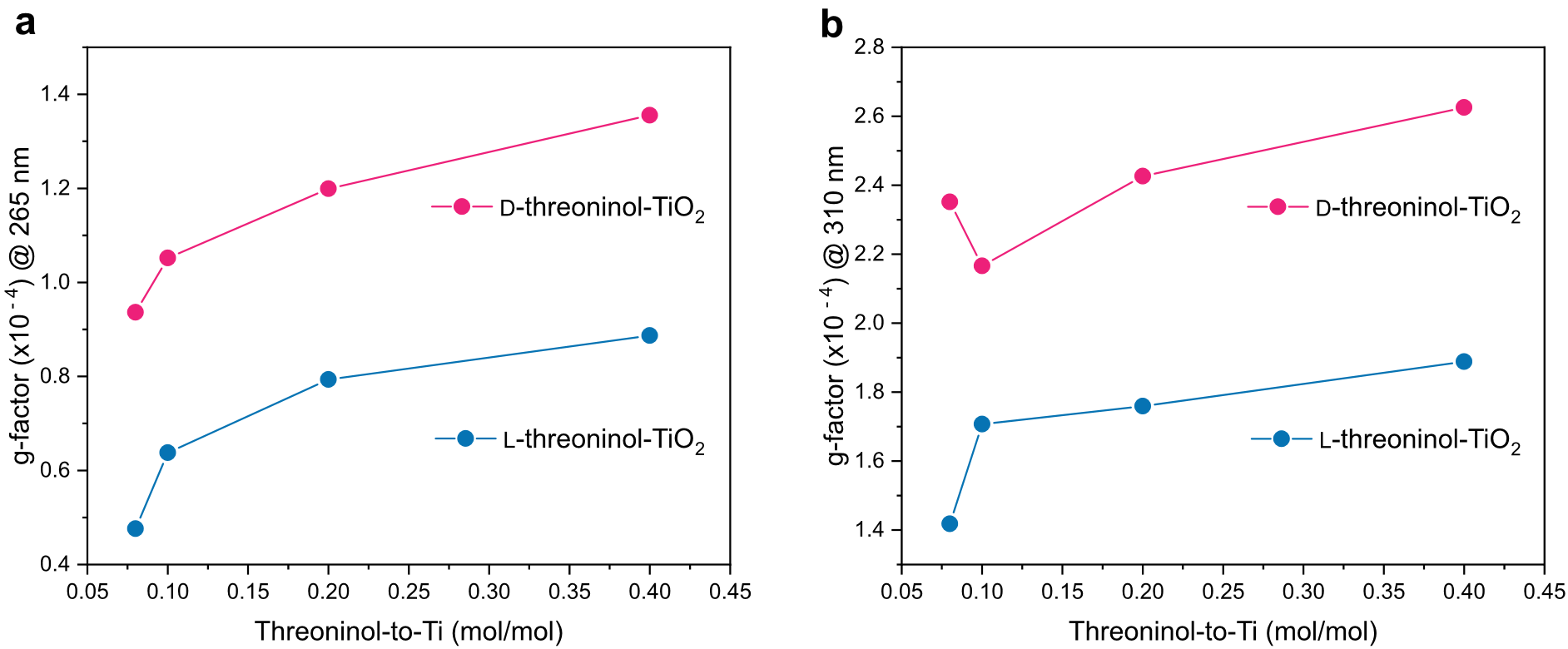


Figure S5. Dependence of g-factor intensity at (a) 265 nm and (b) 310 nm on the threoninol-to-Ti (mol/mol) ratio for l/d-threoninol-functionalized-TiO_2_ NPs. For d-threoninol samples, the g-factor values are shown as absolute values for comparison purposes.


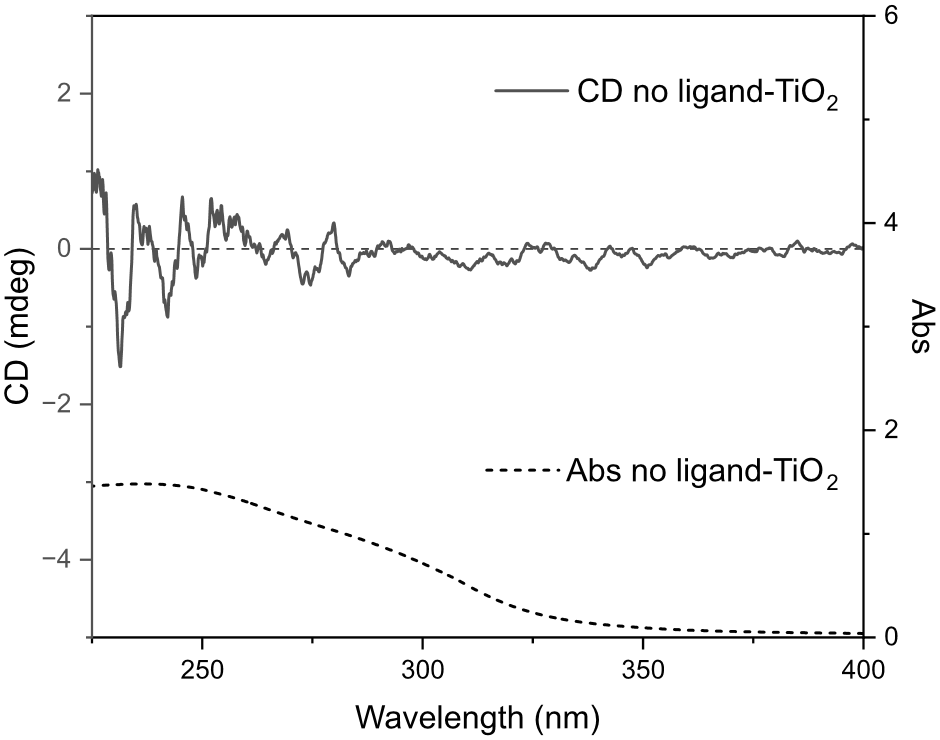


Figure S6. CD spectrum (solid line) and UV-vis spectrum (dashed line) of TiO_2_ NPs synthesized in the absence of a chiral ligand.


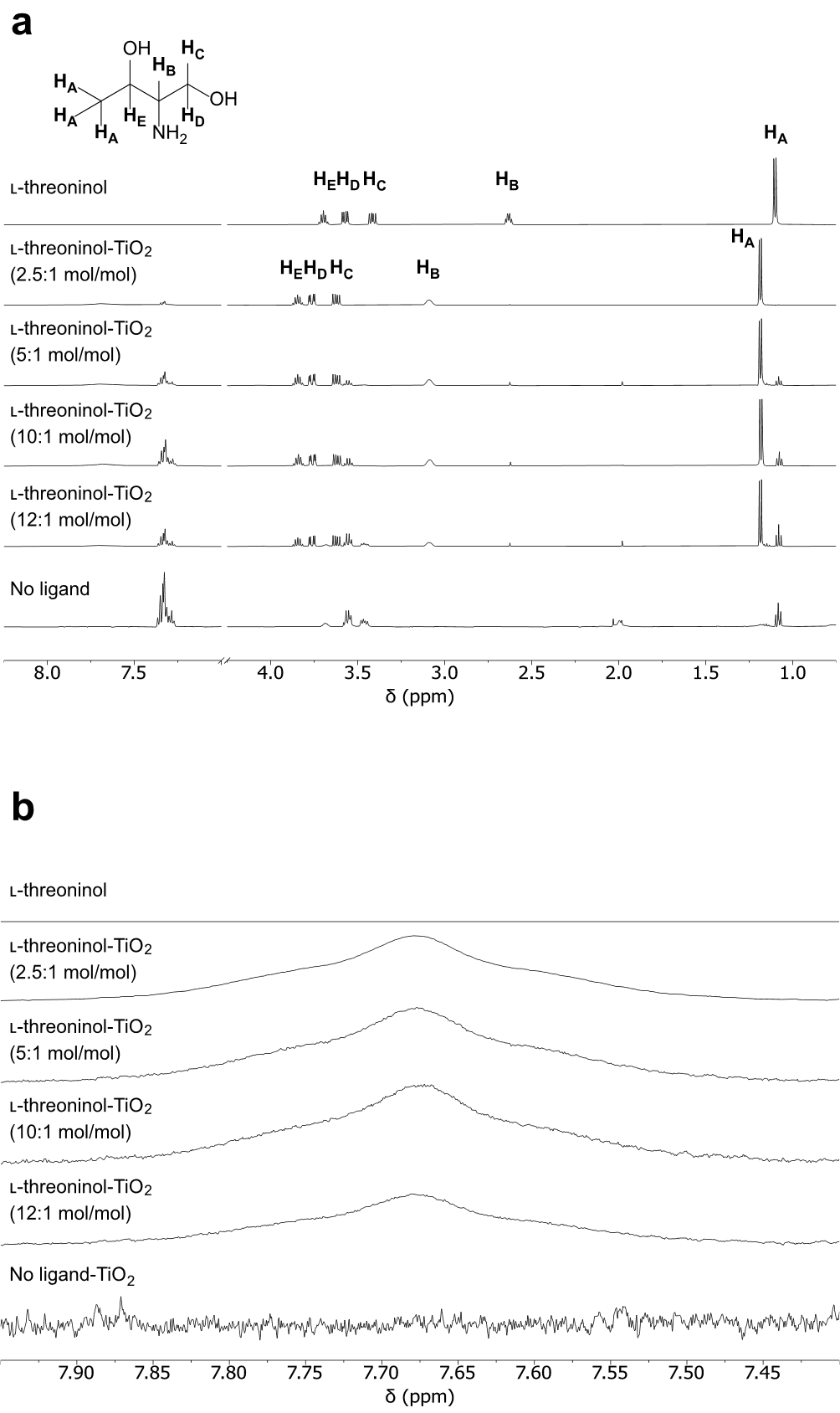


Figure S7. ^1^H NMR spectra of l-threoninol-functionalized TiO_2_ nanoparticles at different Ti-to-threoninol molar ratios (2.5:1, 5:1, 10:1, 12:1), along with spectra of pure l-threoninol and TiO_2_ synthesized without the ligand for comparison. (a) full spectrum, and (b) zoomed-in view of the peak at 7.68 ppm.


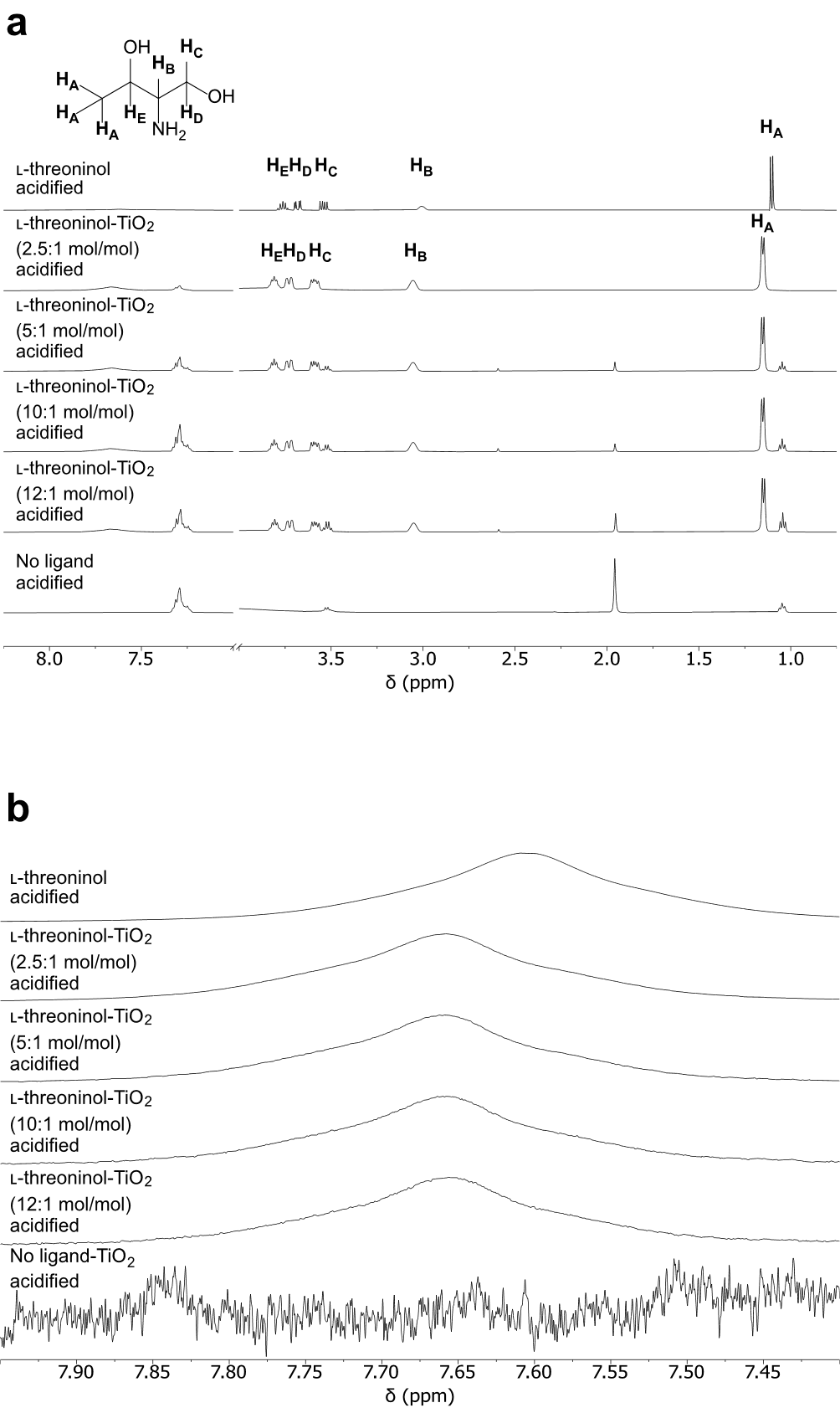


Figure S8. ^1^H NMR spectra of acidified l-threoninol-functionalized TiO_2_ nanoparticles at different Ti-to-threoninol molar ratios (2.5:1, 5:1, 10:1, 12:1), along with spectra of acidified l-threoninol and acidified TiO_2_ synthesized without the ligand for comparison. (a) full spectrum, and (b) zoomed-in view of the peak at 7.68 ppm.


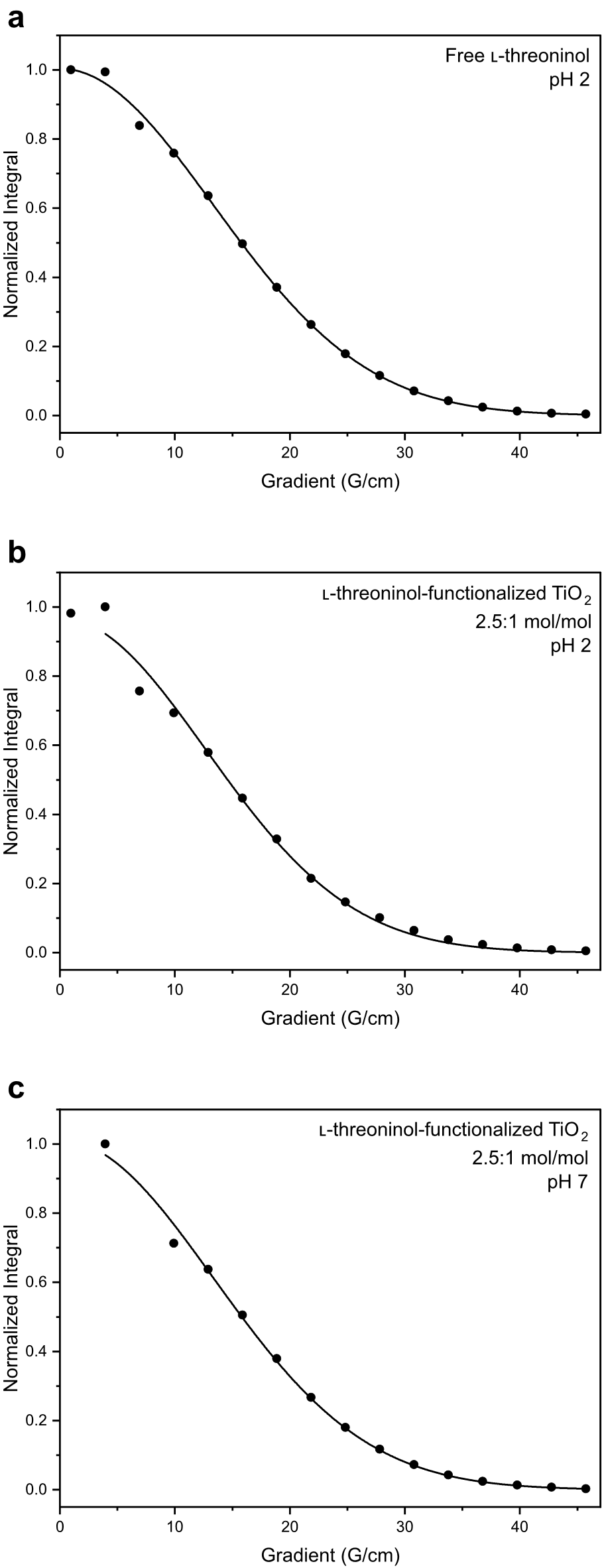


Figure S9. NMR DOSY spectra of (a) pure l-threoninol at pH 2, (b) l-threoninol-functionalized TiO_2_ nanoparticles at a Ti-to-threoninol molar ratio of 2.5:1 at pH 2, and (c) l-threoninol-functionalized TiO_2_ nanoparticles at a Ti-to-threoninol molar ratio of 2.5:1 at pH 7.


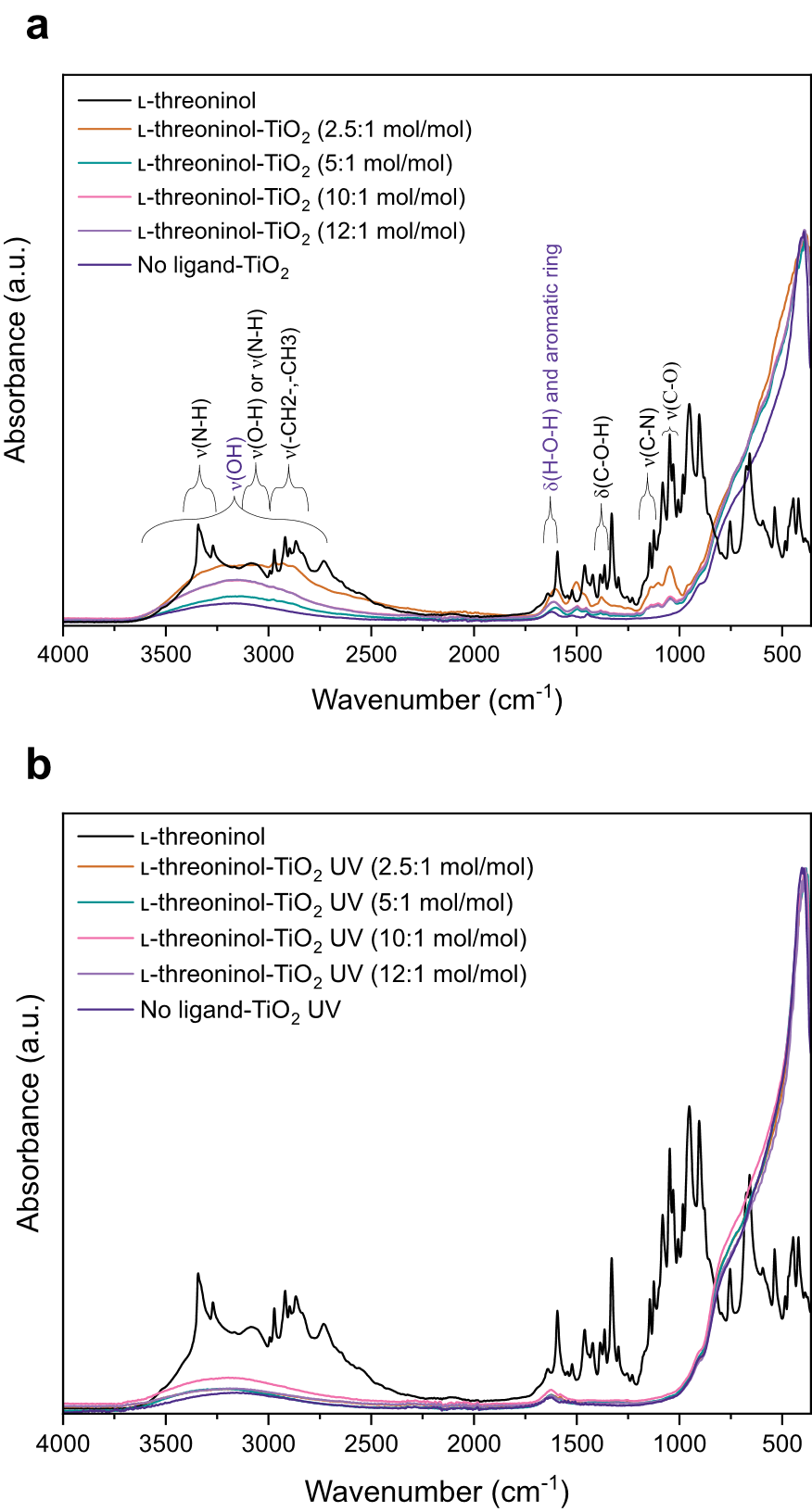


Figure S10. ATR-FTIR spectra of l-threoninol-functionalized TiO_2_ nanoparticles at different Ti-to-threoninol molar ratios (2.5:1, 5:1, 10:1, 12:1), along with spectra of l-threoninol and TiO_2_ synthesized without the ligand for comparison. (a) before, and (b) after UV-treatment.


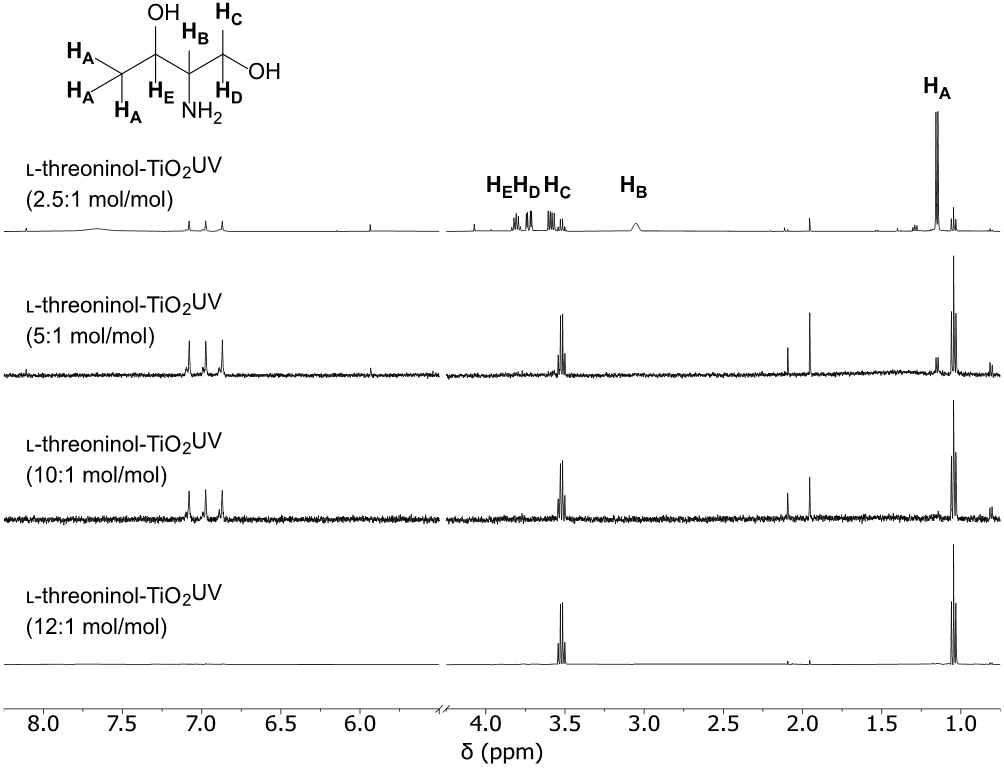


Figure S11. ^1^H NMR spectra of l-threoninol-functionalized TiO_2_ nanoparticles after UV-treatment at different Ti-to-threoninol molar ratios (2.5:1, 5:1, 10:1, 12:1).

Table S2. Average number mean and corresponding polydispersity index for the samples l-threoninol-functionalized TiO_2_ at Ti-to-threoninol molar ratio of 5:1, before and after UV-treatment.


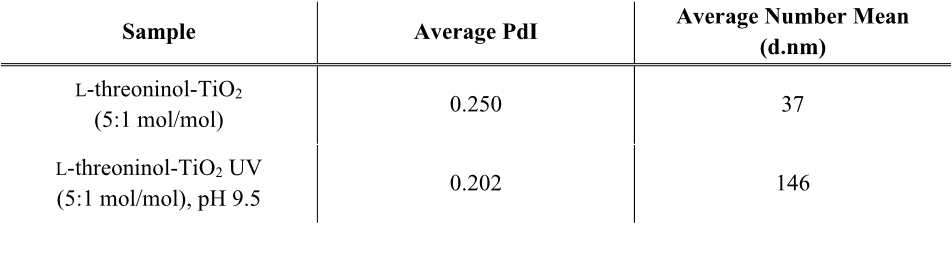


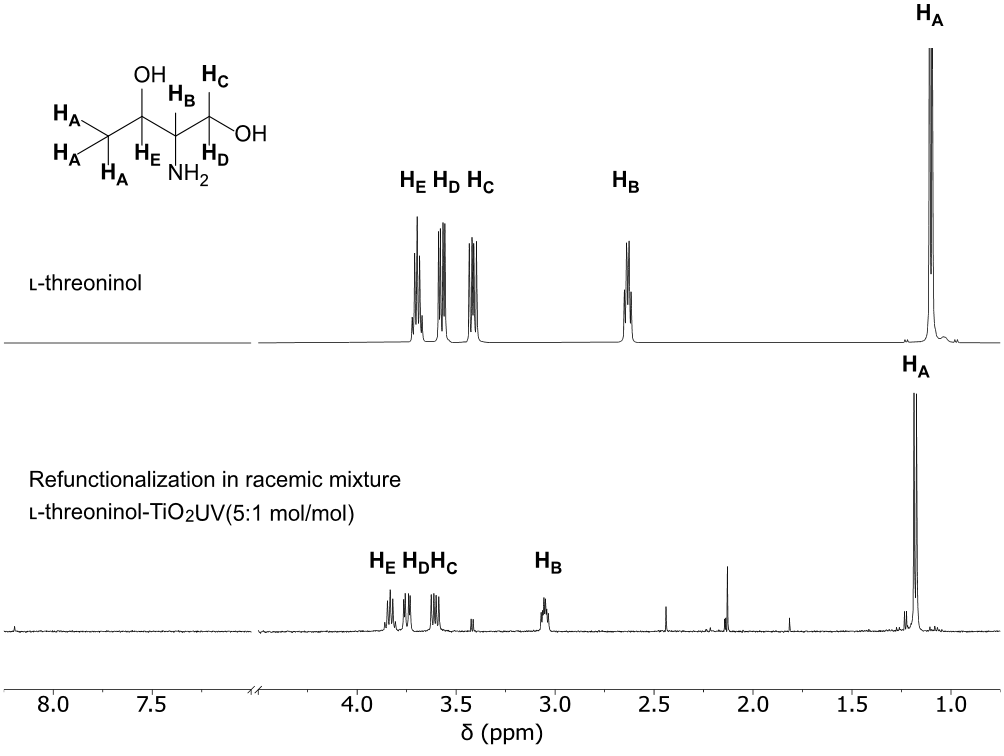


Figure S12. ^1^H NMR spectra of pure l-threoninol (top) and the refunctionalized TiO_2_ nanoparticles in a racemic mixture (bottom).


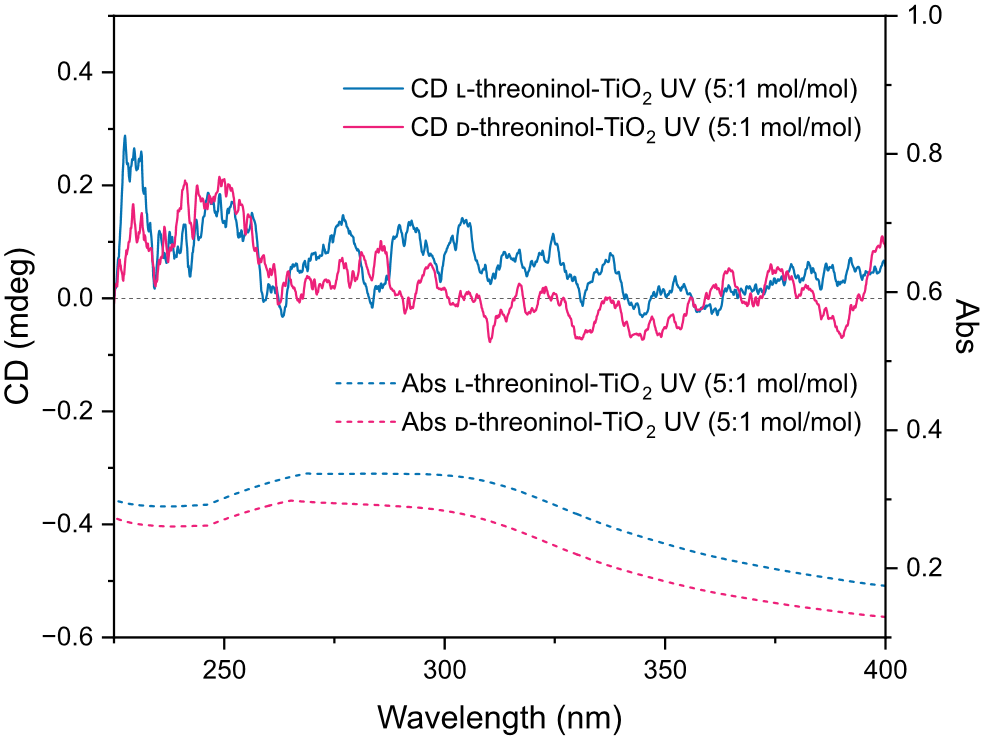


Figure S13. CD spectra (solid lines) and UV-vis spectra (dashed lines) of TiO_2_ nanoparticles initially functionalized with l-threoninol (blue) and d-threoninol (magenta) after UV treatment, stabilized with NaOH at pH ≈9.6. Ti-to threoninol molar ratio of 5:1.

# **References**

[1] J. Polleux, N. Pinna, M. Antonietti, M. Niederberger, *Adv. Mater.* **2004**, *16*, 436-439.

[2] J. Polleux, N. Pinna, M. Antonietti, C. Hess, U. Wild, R. Schlögl, M. Niederberger, *Chem. Eur. J.* **2005**, *11*, 3541-3551.

[3] T. Rajh, L. X. Chen, K. Lukas, T. Liu, M. C. Thurnauer, D. M. Tiede, *J. Phys. Chem. B* **2002**, *106*, 10543-10552.

[4] W. Kuhn, *Trans. Faraday Soc.* **1930**, *26*, 293-308.

[5] R. W. Adams, C. M. Holroyd, J. A. Aguilar, M. Nilsson, G. A. Morris, *Chem. Commun.* **2013**, *49*, 358-360.

[6] M. L. Liu, X. A. Mao, C. H. Ye, H. Huang, J. K. Nicholson, J. C. Lindon, *J. Magn. Reson.* **1998**, *132*, 125-129.

[7] A. Jerschow, N. Muller, *J. Magn. Reson.* **1997**, *125*, 372-375.

[8] E. O. Stejskal, J. E. Tanner, *J. Chem. Phys.* **1965**, *42*, 288–292.
